# Jordans’ anomaly in Chanarin-Dorfman syndrome

**DOI:** 10.1515/almed-2024-0159

**Published:** 2024-11-18

**Authors:** Jorge Sánchez-Cortés, Xavier Gabaldó-Barrios

**Affiliations:** Servicio de Análisis Clínicos, Hospital Universitari Sant Joan de Reus, Reus, Spain

**Keywords:** Jordans’ anomaly, peripheral blood smear, Chanarin-Dorfman syndrome

## Abstract

**Objectives:**

Chanarin-Dorfman syndrome is a rare disease inherited in an autosomal recessive pattern whose prevalence does not exceed 130 cases worldwide.

**Case presentation:**

A 4-year-old patient with generalized erythematous-desquamative ichthyosiform syndrome since birth. The main laboratory finding was persistent hypertransaminasemia. Supplementary studies included peripheral blood smear (PBS), which revealed the presence of multiple cytoplasmatic vacuoles in polymorphonuclear leukocytes (PMN) and platelets. Ichthyosiform lesions concomitant to the presence of lipid vacuoles in peripheral blood PMNs are signs of Chanarin-Dorfman syndrome. Diagnostic suspicion was confirmed by genetic sequencing.

**Conclusions:**

Chanarin-Dorfman syndrome is characterized by a mutation in the *CGI-58* gene. This gene is involved in the catabolism of long-chain triglycerides stored in cytoplasmic lipid droplets. Jordans’ anomaly is a congenital alteration characterized by the presence of multiple vacuoles in the granulocytic series due to defective lipid metabolism. In this syndrome, long-chain triglycerides build up in tissues, thereby causing dermatological manifestations that are controllable through diet.

## Introduction

We report a case of Chanarin-Dorfman syndrome, an extremely rare, recessive autosomal, multisystemic condition characterized by body’s inability to metabolize neutral lipids. It is very infrequent, with a prevalence of less than 130 cases worldwide. Most cases have been detected in countries of the Mediterranean region, especially in Turkey, which may be related to high consanguinity rates [1].

## Case presentation

We report the case of a 4-year-old patient without any familial history of interest referred to our Unit of Dermatology for congenital ichthyosiform syndrome since birth. The patient exhibited erythematous plaques with active scaly edges and involvement of the scalp, face, trunk, elbows, knees, limbs, palms and soles. Skin biopsy revealed hyperkeratosis consistent with seborrheic eczema.

Laboratory tests were unremarkable, except for aspartate aminotransferase (AST), alanine aminotransferase (ALT) and alkaline phosphatase (ALP) (Table 1). Persistent hypertransaminasemia was observed. The clinical morphology of lesions was suggestive of erytrokeratodermia variabilis, given the varying, migratory nature of the hyperkeratotic lesions. However, an abnormal liver profile led physicians to consider other options. The ichthyosiform appearance of the process associated with liver disease led to screening for Chanarin-Dorfman syndrome.

**Table 1:** Results for aminotransferases and alkaline phosphatase.

Magnitude	Result	Reference values
AST	67.2 U/L	0–52 U/L
ALT	67.2 U/L	0–39 U/L
ALP	908.4 U/L	0–269 U/L

ALT, alanine aminotransferase; AST, aspartate aminotransferase; ALP, alkaline phosphatase.

**Figure 1:**
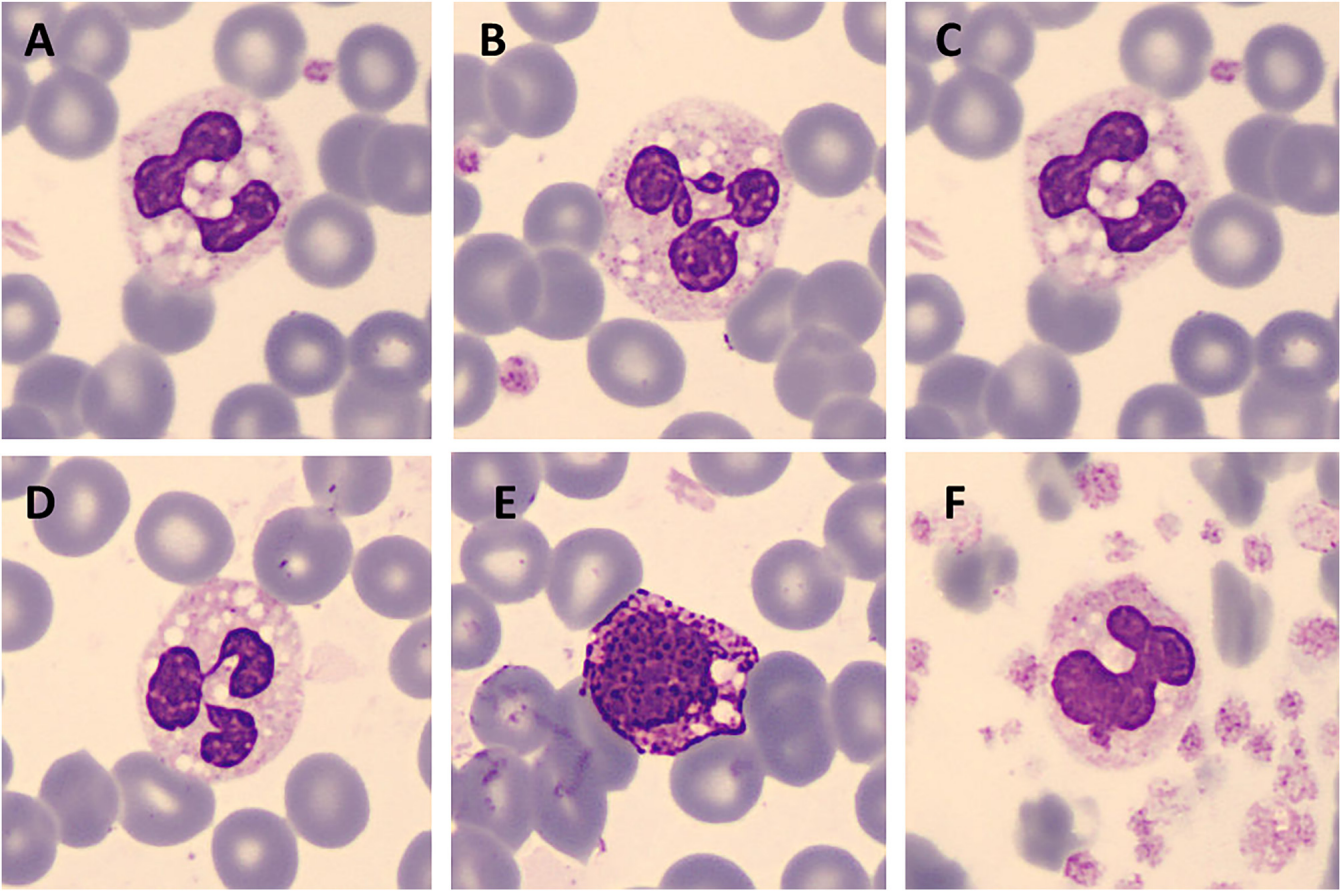
Lipid vacuoles in neutrophils (A–D), basophils (E) and platelets (F). Images acquired with CellaVision^®^ DM96.

Peripheral blood smear (PBS) was revised, revealing the presence of multiple cytoplasmic vacuoles within granular leukocytes and platelets (Figure 1). Generalyzed dry ichthyosiform erythroderma along with the presence of lipid vacuoles in peripheral blood polymorphonuclear cells (PMN) were suggestive of neutral lipid storage disease, also known as Chanarin-Dorfman syndrome. Examination of PBS of the patient’s parents excluded the presence of PMN vacuoles.

Diagnosis was confirmed by Sanger sequencing of the *CGI-58* gene, which causes this syndrome. Genetic testing of the index case and his parents unveiled a nonsense mutation in exon 6 (934G→T; R312X) inherited from the father, and an insertion/deletion at position 617 in exon 4 inherited from the mother. The latter induced a frameshift mutation that caused a premature stop codon. The patient was found to be a trans compound heterozygous carrying both different mutant alleles.

Based on these findings, an ophthalmologic study, a muscle enzyme evaluation, an otorhinolaryngologic study were recommended to screen for potential hearing disorders, as well as a pediatric neurological examination. Additionally, regular dermatological exams were suggested, and the patient was referred to the Nutrition and Dietetics department. The ophthalmological and pediatric neurological exams were unremarkable. However, a mild neurosensorial hearing loss was noted, and creatine kinase levels were 2–5 times the upper limit of normal, albeit the patient did not suffer from muscle pain.

In the absence of a specific treatment for this condition, the only effective treatment available involved restricting dietary long-chain fatty acids and supplementation with medium-chain fatty acids. After 6-month treatment, clinical symptoms improved, with reduced areas of scaly skin and without any significant variations in the levels of AST, ALT and ALP.

## Discussion

Jordans was the first to describe the presence of abundant non-polar vacuoles in varying size in leukocytes on the PBS of two brothers suffering from progressive muscular dystrophy. Vital staining by the Casaris-Demel method (mixture of brilliant cresyl blue, Sudan III and absolute alcohol) revealed a red inclusion in the cytoplasm of most neutrophils, eosinophils and basophils, which confirmed the lipidic nature of the vacuoles. Glycogen could not be detected in the vacuoles [2]. For this reason, this trait is known as Jordan’s anomaly. In 1974, Dorfman et al. reported the case of a 35-year-old male patient with white scales on skin concomitant to Jordans’ anomaly. A year later, Charain et al. reported a similar case in a 22 year-old female. Deposition of lipids in the liver added to the absence of this anomaly in the family raised strong suspicion of a systemic syndrome characterized by a defective lipid metabolism [3], 4].

Chanarin-Dorfman is a rare syndrome characterized by ichthyosis and the presence of multiple cytoplasmic lipid vacuoles in several tissues, predominantly in peripheral blood granulocytes, the skin and the liver [5]. This syndrome is associated with a wide range of clinical symptoms, with all patients suffering from congenital ichthyosiform erythroderma and hepatic steatosis. Other organs may be variably involved, including the eyes, ears and muscles. Varying symptoms include bilateral ectropion, cataracts, strabismus, nystagmus, alopecia, microtia with bilateral sensorineural deafness, hepatosplenomegaly, and in some rare cases, intellectual disability and short stature. These symptoms may vary according to the ethnicity and mutations of the patients [1], 5]. The concomitant presence of Jordans’ anomaly in several tissues and ichthyosis are clinical traits commonly used for establishing diagnosis of Chanarin-Dorfman.

In this syndrome, lipid droplets are mostly observed in the cytoplasm of blood cells. However, these vacuoles are also found in biopsies of the skin, the bone marrow and the liver and, less frequently, in the small bowel, the stomach and the kidneys [6], [7], [8]. Moreover, in the rare cases where Jordans’ anomaly is not present in PBS cells, the accumulation of lipid vacuoles is observed in skin biopsies by transitional electron microscopy [9].

Lipid droplets play a major role in energy homeostasis. These cytoplasmic vacuoles store excess energy in the form of triacylglycerols and, in the case of energy deficit, triacylglycerols are hydrolyzed to glycerol and fatty acids. The *ABHD5* (α/β-hydrolase domain-containing 5), also known as *CGI-58* (comparative gene identification-58), is a member of an α/β-hydrolase domain protein family that is located in chromosome 3p21.33. This protein consists of 349 amino acids with a molecular mass of approximately 39 kD and is observed on the surface of the lipid droplets of the cytoplasm [10].

*ABHD5* acts as a coactivator of the adipose triglyceride lipase (*ATGL*), which is a key enzyme in the regulation and initiation of the hydrolysis of cytoplasmic lipid droplets [10]. As it occurs in *ABHD5*, *ATGL* mutations cause a neutral lipid storage disease, with the accumulation of triglyceride-containing cytoplasmic lipid droplets in multiple tissues [11]. Unlike Chanarin-Dorfman, which causes ichthyosis, it is associated with myopathy.

Ichthyosis is a disorder associated with skin permeability barrier defects. Ichthyosiform erythroderma is characterized by the appearance of thin white scales on an erythematous surface all over the body, except in skin folds of the limbs, where active desquamative plaques are predominant [12]. In healthy skin, the space between the stratum corneum and the stratum granulosum is occupied by lipid lamellar membranes that ensure the structural protective role of the skin. These lipids are primarily composed of ceramides, cholesterol and free fatty acids. *ATGL* is involved in the synthesis of acylceramide, a key component of the lipid lamellae surrounding corneocytes, which is essential for maintaining the structure of the epidermis [13]. Therefore, acylceramide production is impaired in carriers of *ABHD5* mutations, thereby inducing separation of the lamellar/non-lamellar phases and originating a dry, squamous skin.

There are no therapies currently available for Chanarin-Dorfman. The only effective treatment involves restricting dietary long-chain fatty acids. Additionally, there is evidence that diet rich in medium-chain fatty acids improve skin lesions. However, levels of aminotransferases keep elevated over time [14], 15]. Acitretin, a second-generation retinoid can also improve desquamation and erythema and significantly reduce the extent of lesions [10].

## Lessons learned

– Jordans’ anomaly in peripheral blood leukocytes after staining with May-Grünwald Giemsa is the most frequent laboratory finding in neutral lipid storage disease.
– All cases of Chanarin-Dorfman syndrome exhibit Jordans’ anomaly and ichthyosis.
– Restricted dietary intake of long-chain fatty acids is essential for an appropriate control of neutral lipid storage diseases.
